# Survival of patients with symptom- and screening-detected colorectal cancer

**DOI:** 10.18632/oncotarget.9412

**Published:** 2016-05-17

**Authors:** Hermann Brenner, Lina Jansen, Alexis Ulrich, Jenny Chang-Claude, Michael Hoffmeister

**Affiliations:** ^1^ Division of Clinical Epidemiology and Aging Research, German Cancer Research Center (DKFZ), Heidelberg, Germany; ^2^ Division of Preventive Oncology, German Cancer Research Center (DKFZ), Heidelberg, Germany; ^3^ German Cancer Consortium (DKTK), German Cancer Research Center (DKFZ), Heidelberg, Germany; ^4^ Department of General, Visceral and Transplantation Surgery, University of Heidelberg, Heidelberg, Germany; ^5^ Unit of Genetic Epidemiology, German Cancer Research Center (DKFZ), Heidelberg, Germany

**Keywords:** colonoscopy, colorectal cancer, fecal occult blood test, screening, survival

## Abstract

**Background:**

An increasing proportion of colorectal cancer (CRC) patients are diagnosed by screening rather than symptoms.

**Aims:**

We aimed to assess and compare prognosis of patients with screen-detected CRC and symptom-detected CRC.

**Methods:**

Overall and CRC specific mortality over a median follow-up of 4.8 years was assessed according to mode of diagnosis (symptoms, screening colonoscopy, fecal occult blood test [FOBT], other) in a multi-center cohort of 2,450 CRC patients aged 50-79 years recruited in Germany in 2003-2010.

**Results:**

68%, 11% and 10% were detected by symptoms, screening colonoscopy and FOBT, respectively. The screen-detected cancers had a more favorable stage distribution than the symptom-detected cancers (68% versus 50% in stage I or II). Age- and sex adjusted hazard ratios (HRs) of total mortality with 95% confidence intervals (95% CIs) compared to symptom-detected cancers were 0.35 (0.24-0.50) and 0.36 (0.25-0.53) for screening colonoscopy and FOBT detected CRCs, respectively. HRs were only slightly attenuated and remained highly significant after adjustment for stage and multiple other covariates (0.50 (0.34-0.73) and 0.54 (0.37-0.80), respectively). Even stronger associations were seen for CRC specific mortality. Patients with screen-detected stage III CRC had as good CRC specific survival as patients with symptom-detected stage I or II CRC.

**Conclusions:**

Patients with screen-detected CRC have a very good prognosis far beyond the level explained by their more favorable stage distribution. Mode of detection is an important, easy-to-obtain proxy indicator for favorable diagnosis beyond earlier stage at diagnosis and as such may be useful for risk stratification in treatment decisions.

## INTRODUCTION

Screening for colorectal cancer (CRC) is employed in an increasing number of countries due to its proven efficacy in reducing CRC incidence and mortality [1–9], and an increasing share of CRC cases is diagnosed by screening rather than by symptoms. It is to be expected that patients with screen-detected CRC have better prognosis than patients with symptom-detected CRC due to earlier diagnosis and potentially other favorable tumor or host characteristics. However, it is unclear to what extent survival advantages persist within stages at diagnosis. Direct evidence on overall and stage-specific survival expectations of patients with screen-detected CRC is surprisingly sparse. Such evidence would though be of major clinical and public health interest for several reasons: First, it may alleviate the fear of having a diagnosis of CRC at screening which may be a major obstacle for people to undergo CRC screening. Second, it may alleviate fears of patients after a CRC diagnosis at screening. Third, it may enhance the basis for clinical decision making.

In this manuscript, we provide a detailed assessment of prognosis of CRC patients according to type of diagnosis (detection by symptoms, screening colonoscopy, fecal occult blood test [FOBT], or otherwise) in a large multi-center cohort of CRC patients from Germany for whom detailed information on the diagnostic process was obtained.

## MATERIALS AND METHODS

### Study design and study population

In Germany, screening colonoscopy is offered to the average risk population from age 55 on. Screening by (guaiac based) FOBT is offered annually at ages 50 to 54. From age 55 on, FOBT every two years is offered as an alternative to screening colonoscopy. To assess the impact of screening, the DACHS (Darmkrebs: Chancen der Verhütung durch Screening) study was initiated in the Rhine-Neckar area of Germany in 2003. DACHS is a population-based case-control study with additional comprehensive follow-up of cases. Details of the study design and data collection have been reported previously [10–12]. Briefly, patients with a first diagnosis of CRC (ICD 10 codes C18-C20) aged 30 years or older are recruited in all of the 22 hospitals providing CRC surgery in the study region (approximately 2 million inhabitants). Matched controls are randomly selected from population registries. The study was approved by the ethics committees of the Medical Faculty of the University of Heidelberg and of the Medical Chambers of Baden-Württemberg and Rhineland-Palatinate. Written informed consent is obtained from each participant. Participants for the current analysis were selected from 3,146 cases recruited from 2003 to 2010 and followed with respect to survival to 2013.

### Data collection

Personal interviews with cases were conducted by trained interviewers using a standardized questionnaire. Interviews were conducted during hospital stay, typically a few days after surgery, wherever possible, or after hospital discharge at the patients’ homes otherwise. In addition, medical data were extracted from hospital charts. The interviews lasted for about one hour and addressed potential CRC risk factors, preventive factors and prognostic factors in great detail. In addition, detailed information was collected on history of CRC screening and the basis for the current CRC diagnosis. In particular, patients were asked if the diagnosis was made by work-up of symptoms, by a screening examination or incidentally (e.g. in the context of medical examination for other reasons). If the cancer was detected by screening, the type and sequence of screening examinations was ascertained.

Data extracted from medical charts include tumor stage and location in particular. Three years after diagnosis, standardized information on CRC therapy was obtained from the physicians of the patients. Three and 5 years after diagnosis, vital status was ascertained through systematic follow-up by record linkage with population registries. For deceased patients, cause of death was extracted from death certificates which were obtained from local public health authorities.

### Statistical analysis

For statistical analysis, the following consecutive exclusions were made (numbers and reasons given in parentheses): <50 or >=80 years of age (n=664; screening not commonly offered or recommended for the average risk population at these ages), history of inflammatory bowel disease (n=19; frequent surveillance colonoscopies due to increased risk of CRC), missing information on the mode of CRC detection (n=2), and missing follow-up data (n=11). After these exclusions, there remained 2,450 cases for the analysis.

We first described cases according to age, sex, education, stage (using the Union Internationale Contre le Cancer [UICC] classification), site (proximal colon, cecum to transverse colon; distal colon, left flexure to sigmoid colon; rectum) and mode of detection (symptoms; screening colonoscopy; FOBT; other, such as incidental detection in the course of other diagnostic measures) of the cancer. Differences in age, sex, education, stage, and site distributions by mode of detection were assessed by Chi square tests.

Next, we assessed overall and CRC specific survival according to mode of detection. Survival time was calculated from the day of diagnosis to the day of death (deceased patients) or censored at the date of the last follow-up. In cause specific survival analyses survival time was censored at the day of death from other reasons.

Survival was compared by mode of detection using direct adjusted survival curves (adjusted for age and sex). In addition, Cox proportional hazards models were run to evaluate the association of mode of detection with survival outcomes using various levels of adjustment: Model 1 adjusted for sociodemographic variables only (sex, age, education). Model 2 additionally adjusted for key tumor characteristics, i.e., stage and location. In order to assess potential variation of results according to specific treatments, Cox proportional hazards models were repeated after excluding 304 patients who had received neoadjuvant therapy in sensitivity analyses. Furthermore, we carried out specific analyses on CRC specific survival for subgroups defined by tumor stage and location, by conduct of chemotherapy among stage II and stage III patients, and by sex and age. In all Cox models, the proportional hazards assumption was checked by testing for interaction of the covariates with follow-up time and interaction terms were added as needed.

All statistical analyses were carried out using SAS statistical software, version 9.3 (SAS Institute Inc., Cary, North Carolina). An alpha level of 0.05 was employed for statistical tests.

## RESULTS

Table 1 shows characteristics of the study population which included 1520 (62%) male and 930 (38%) female patients. The majority of patients were between 60 and 79 years old (82%; median: 68 years), were diagnosed at stage II or III (62%), and had their cancer detected by symptoms (68%). Screening colonoscopy, FOBT and other reasons led to the diagnosis in approximately 10% each.

**Table 1:** Characteristics of the study population

Characteristic	Men		Women		Total	
	n	%	N	%	n	%
*Age*						
50-59 years	273	18%	175	19%	448	18%
60-69 years	636	42%	363	39%	999	41%
70-79 years	611	40%	392	42%	1003	41%
*Education^a^*						
≤9 years	1057	70%	651	70%	1708	70%
10-11 years	204	13%	178	19%	382	16%
12+ years	259	17%	97	11%	356	14%
*Cancer stage^b^*						
I	365	24%	210	23%	575	23%
II	459	30%	289	31%	748	31%
III	460	31%	304	33%	764	31%
IV	231	15%	125	13%	356	15%
*Cancer site*^c^						
Proximal colon	399	26%	332	36%	731	30%
Distal colon	419	28%	268	29%	687	28%
Rectum	699	46%	327	35%	1026	42%
*Mode of detection*						
Symptoms	997	66%	673	73%	1670	68%
Screening colonoscopy	183	12%	96	10%	279	11%
FOBT	168	11%	75	8%	243	10%
Other	172	11%	86	9%	258	11%

FOBT, fecal occult blood test

a Information missing for 4 patients.

b Information missing for 7 patients.

c Information missing for 6 patients.

Patients whose cancer was detected by screening colonoscopy more often had higher education than patients with symptom-detected cancer (Table 2). Furthermore, their cancer was detected much more often in stage I (50% versus 17%), and located in the colon (69% versus 53%). FOBT detected cancers also had a more favourable stage distribution than symptom-detected cancers, with only 4% of cancers detected in stage IV, compared to 17% for symptom-detected cancers and 5% for screening colonoscopy-detected cancers. However, the proportion of stage I cancers was lower than among screening colonoscopy-detected cancers.

**Table 2:** Association of mode of detection with patient and tumor characteristics

Characteristic	Mode of detection										
	Symptoms		Screening colonoscopy		p-value^a^	FOBT		p-value ^a^	Other		p-value ^a^
	n	%	n	%		n	%		n	%	
*Age*											
50-59	322	19%	40	14%	0.037	47	19%	0.43	39	15%	0.066
60-69	679	41%	134	48%		89	37%		97	38%	
70-79	669	40%	105	38%		107	44%		122	47%	
*Sex*											
Men	997	60%	183	66%	0.062	168	69%	0.005	172	67%	0.033
Women	673	40%	96	34%		75	31%		86	33%	
*Education*											
≤9 years	1189	71%	175	63%	0.003	163	67%	0.262	181	67%	0.773
10-11 years	258	15%	47	17%		39	16%		38	16%	
12+ years	220	13%	57	20%		41	17%		38	17%	
*Cancer stage*											
I	291	17%	139	50%	<0.001	83	34%	<0.001	62	24%	0.048
II	545	33%	47	17%		82	34%		74	29%	
III	547	33%	76	27%		67	28%		74	29%	
IV	283	17%	15	5%		10	4%		48	18%	
*Cancer* site											
Prox. colon	428	26%	97	35%	<0.001	88	36%	<0.001	118	46%	<0.001
Distal colon	449	27%	96	34%		72	30%		70	28%	
Rectum	790	47%	86	31%		83	34%		67	26%	

FOBT, fecal occult blood test

a p-value for difference from participants whose cancer was detected by symptoms

**Figure 1:**
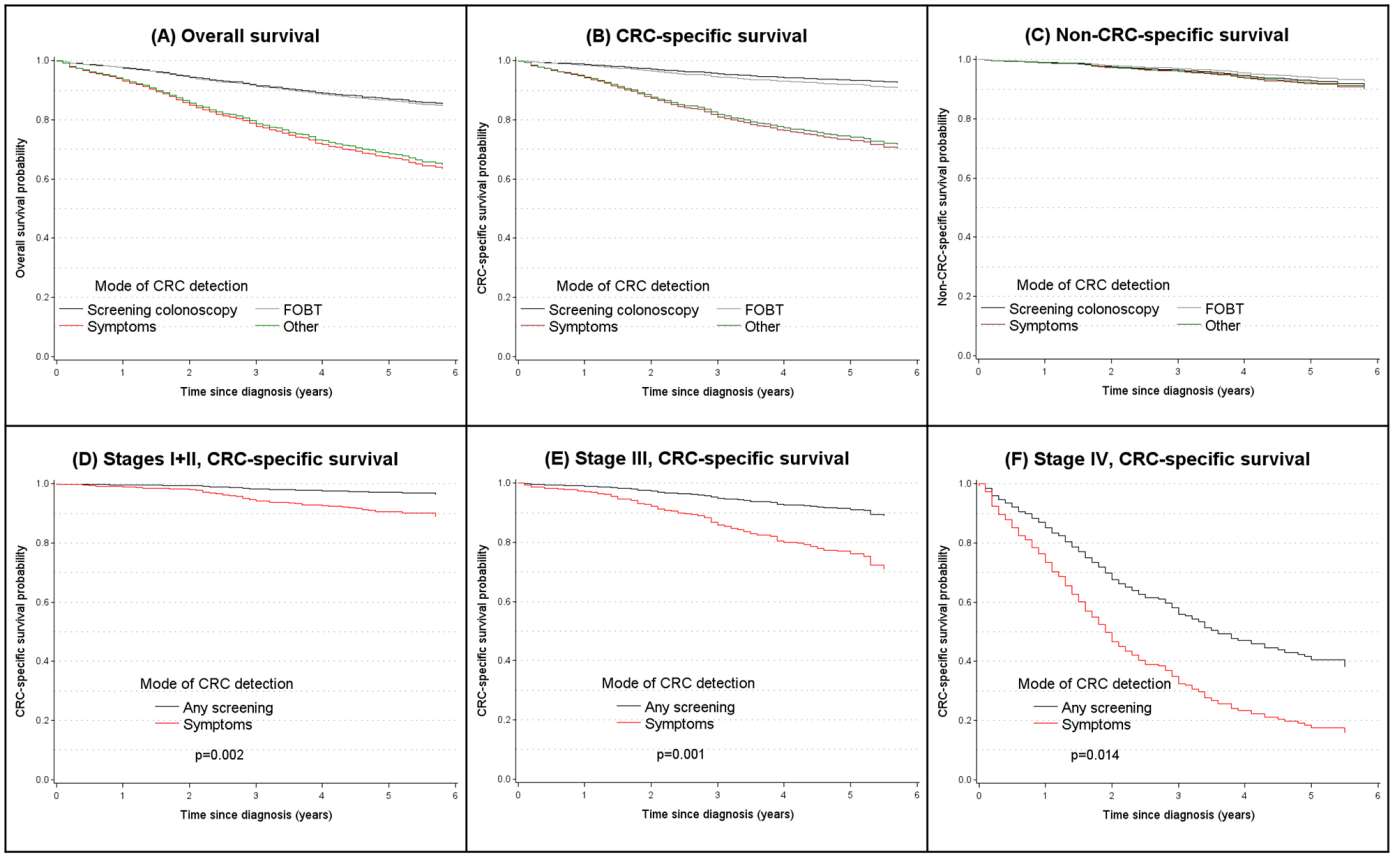
Direct adjusted survival curves according to mode of cancer detection for A overall and **B.** CRC-specific survival, **C.** non-CRC-specific survival and **D, E, F.** according to mode of detection and stage at diagnosis (all survival curves were adjusted for age and sex).

During a median follow-up time of 4.8 years (interquartile range: 3.0 to 5.1 years), 590 (24%) patients died, of whom 461 (78%) died from CRC. Direct adjusted survival curves are shown in Figure 1. Patients whose cancer was detected by screening colonoscopy or FOBT had substantially higher overall survival and CRC specific survival 5 years after diagnosis (>85% and >90%, respectively) than patients whose cancer was detected by symptoms or otherwise (<70% and <75%, respectively). After adjustment for age, sex and education, patients whose cancer was detected by screening colonoscopy had a 65% lower total mortality than patients with symptom-detected cancers (hazard ratio, HR, 0.35, 95% confidence interval, 95% CI, 0.24-0.50) (Table 3). A 50% mortality reduction persisted even after additional control for stage and location of the cancer (HR 0.50, 95% CI 0.34-0.73). Similarly decreased total mortality was seen for patients whose cancer was detected by FOBT. Even stronger reductions were seen for CRC mortality, with fully adjusted hazard ratios of 0.36 (95% CI 0.21-0.60) and 0.47 (95% CI 0.29-0.77) for patients with screening colonoscopy and FOBT-detected cancers, respectively. By contrast, no difference was seen in mortality from other causes between patients with screen-detected and symptom-detected cancer. Sensitivity analyses excluding 304 patients who had received neoadjuvant therapy did not materially change any of the observed associations (fully adjusted HRs (95% CIs) for CRC mortality: 0.38 (95% CI 0.23-0.64) and 0.48 (95% CI 0.29-0.79) for colonoscopy- and FOBT detected cancers, respectively). The same applies to sensitivity analyses specifically adjusting for T- and N-status in addition to UICC stage (fully adjusted HRs (95% CIs) for CRC mortality: 0.37 (95% CI 0.21-0.64) and 0.52 (95% CI 0.31-0.85) for colonoscopy- and FOBT detected cancers, respectively).

**Table 3:** Hazard ratios for total, CRC specific and other mortality according to mode of detection

Outcome, mode of detection	Mortality			Hazard Ratios			
	Deaths	Person-years	Rate per 1000 person-yrs	Adjusted for age, sex, education ^a^		Fully adjusted ^b^	
				HR	95% CI	HR	95% CI
*Total mortality*							
Symptoms	461	5815	79.3	1.00	Ref.	1.00	Ref.
Screening colonoscopy	30	1073	28.0	0.35	(0.24-0.50)	0.50	(0.34-0.73)
FOBT	29	971	29.9	0.36	(0.25-0.53)	0.54	(0.37-0.80)
Other	70	899	77.9	0.94	(0.73-1.21)	0.86	(0.66-1.11)
*CRC mortality*							
Symptoms	373	5815	64.1	1.00	Ref.	1.00	Ref.
Screening colonoscopy	15	1073	14.0	0.21	(0.13-0.36)	0.36	(0.21-0.60)
FOBT	17	971	17.5	0.27	(0.17-0.44)	0.47	(0.29-0.77)
Other	56	899	62.3	0.93	(0.70-1.23)	0.85	(0.63-1.13)
*Non CRC mortality*							
Symptoms	88	5815	15.1	1.00	Ref.	1.00	Ref.
Screening colonoscopy	15	1073	14.0	0.92	(0.53-1.59)	0.94	(0.53-1.67)
FOBT	12	971	12.4	0.73	(0.40-1.33)	0.74	(0.40-1.37)
Other	14	899	15.6	0.99	(0.56-1.74)	0.96	(0.54-1.72)

FOBT, fecal occult blood test; HR, hazard ratio, 95% CI, 95% confidence interval

a Adjusted for age, sex, and education, and for a time-dependent effect of age and sex.

b Additionally adjusted for stage and location of the cancer, and for time-dependent effects of stage and location.

Given very similar results for survival of patients with screening colonoscopy and FOBT-detected cancers both groups were combined in further, subgroup specific analyses. Strongly reduced CRC specific mortality for patients with screening colonoscopy or FOBT-detected compared to symptom-detected cancers was consistently seen even after full adjustment for all subgroups defined by tumor stage or location (Table 4, Figure 1, Panels D, E, F), sex or age (Table 5). With a 74% lower CRC specific mortality for screen-detected compared to symptoms-detected cancers, the association was particularly strong among stage II-III patients who received chemotherapy. Additional subgroup analyses among stage III patients by number of affected lymph nodes (N1: 1-3 lymph nodes, N2: 4+ lymph nodes) confirmed substantially lower CRC mortality for patients with screen-detected CRC than for patients with symptom-detected CRC within both subgroups (N1: HR 0.34, 95% CI 0.14-0.81; N2: HR 0.39, 95% CI 0.40-1.10). Among patients with screen-detected stage III CRC, 5-year CRC specific survival was as high (slightly above 90%) as among patients with symptom-detected stage I or II CRC (Figure 1, Panels D and E). The small number of patients with screen-detected stage IV CRC had approximately two-fold higher 5-year CRC-specific survival (40% versus 20%) and 2-fold longer median CRC-specific survival (4 versus 2 years) compared to patients with symptom-detected stage IV CRC (Figure 1, Panel F).

**Table 4:** Hazard ratios for CRC-specific mortality according to mode of detection and stage at diagnosis and location of tumor

Patient / tumor characteristics, mode of detection	Deaths	Person-years	Rate per 1,000 person-yrs	Fully adjusted hazard ratio ^a^	
				HR	95% CI
*Stage I + II*					
Symptoms	60	3225	18.6	1.00	Ref.
Any screening	8	1410	5.7	0.29	(0.14-0.61)
Other	5	528	9.5	0.46	(0.18-1.17)
*Stage III*					
Symptoms	102	1983	51.4	1.00	Ref.
Any screening	10	547	18.3	0.33	(0.17-0.64)
Other	14	266	52.6	0.86	(0.47-1.57)
*Stage IV*					
Symptoms	209	597	350.1	1.00	Ref.
Any screening	14	72	194.4	0.52	(0.30-0.90)
Other	37	105	352.4	0.92	(0.64-1.31)
*Stage II-III patients who received chemotherapy^b^*					
Symptoms	98	2452	40.0	1.00	Ref.
Any screening	7	583	12.0	0.26	(0.12-0.56)
Other	11	270	40.7	0.69	(0.35-1.36)
*Stage II-III patients who did not receive chemotherapy*					
Symptoms	84	2443	34.4	1.00	Ref.
Any screening	10	621	16.1	0.45	(0.23-0.87)
Other	7	312	22.4	0.67	(0.30-1.48)
*Proximal colon*					
Symptoms	112	1380	81.2	1.00	Ref.
Any screening	11	721	15.3	0.32	(0.17-0.60)
Other	22	431	51.0	0.65	(0.40-1.04)
*Distal colon*					
Symptoms	91	1627	55.9	1.00	Ref.
Any screening	10	670	14.9	0.47	(0.24-0.93)
Other	20	225	88.9	1.40	(0.85-2.31)
*Rectum*					
Symptoms	168	2799	60.0	1.00	Ref.
Any screening	11	653	16.8	0.47	(0.25-0.86)
Other	14	230	60.9	0.70	(0.40-1.22)

FOBT, fecal occult blood test; HR, hazard ratio; 95% CI, 95% confidence interval

a Adjusted for age, sex, education, stage and location of the cancer, and for time-dependent effects of age, sex, stage and location.

b Colon cancer patients who received chemotherapy after surgery for treatment of primary cancer; rectum cancer patients who received neoadjuvant therapy before surgery and/or chemotherapy after surgery for treatment of primary cancer.

**Table 5:** Hazard ratios for CRC-specific mortality according to mode of detection and by age and sex

Patient characteristics, mode of detection	Deaths	Person-years	Rate per 1,000 person-yrs	Fully adjusted hazard ratio ^a^	
				HR	95% CI
*Men*					
Symptoms	226	3420	66.1	1.00	Ref.
Any screening	21	1371	15.3	0.38	(0.24-0.59)
Other	34	592	57.4	0.75	(0.52-1.08)
*Women*					
Symptoms	147	2395	61.4	1.00	Ref.
Any screening	11	672	16.4	0.45	(0.24-0.83)
Other	22	307	71.7	1.05	(0.66-1.67)
*Age ≤68 years*^b^					
Symptoms	212	3574	59.3	1.00	Ref.
Any screening	15	1231	12.2	0.38	(0.22-0.65)
Other	24	499	48.1	0.72	(0.46-1.12)
*Age >68 years*^b^					
Symptoms	161	2241	71.8	1.00	Ref.
Any screening	17	812	20.9	0.43	(0.26-0.72)
Other	32	400	80.0	0.92	(0.63-1.35)

FOBT, faecal occult blood test; HR, hazard ratio. 95% CI, 95% confidence interval

a Adjusted for age, sex, education, stage and location of the cancer, and for time-dependent effects of age, sex, stage and location.

b Age categorized at the median value

## DISCUSSION

In this large cohort of patients with CRC recruited in the context of a population-based case-control study in Southern Germany, patients whose cancer was detected by screening colonoscopy or FOBT had strongly enhanced overall survival and even more strongly enhanced CRC specific survival compared to patients with symptom-detected cancer. Even though survival differences were partly explained by the more favourable stage distribution of patients with screening detected cancers, the largest share of the survival advantages persisted even after control for CRC stage, and large survival advantages were seen within each stage. Patients with screen-detected stage III CRC had as good CRC specific survival as patients with symptom-detected stage I or II CRC.

Our findings of more favourable stage distribution among screen-detected cancers than among symptom-detected cancers are consistent with previous reports from various countries including Germany [e.g. 13–17]. Higher survival rates of patients with screen-detected CRC than of patients with symptom-detected CRC have also been repeatedly reported [16–25], but few studies have addressed survival by stage, and the survival advantage has primarily been attributed to more favorable stage distribution. However, in agreement with our findings, Mapp et al found a significant survival advantage in patients with screen-detected cancers in the Nottingham FOBT trial which persisted after control for tumor stage [19]. In two studies (n=633 and 514, respectively) conducted in the context of the FOBT based British Bowel Cancer Screening Programme, survival advantages for screen-detected cancers over symptom-detected cancers were likewise observed even in stage-specific and stage-adjusted analyses [22, 24]. Similar results were recently reported for screening colonoscopy detected colon cancer in a single center study (n=1,071) from the US [25]. In our multi-center study from Germany (n=2,450), we observed similarly strong survival advantages for patients with either FOBT or screening colonoscopy-detected CRC which were only reduced to a small extent and remained highly statistically significant after control for stage in multivariable analysis.

Higher survival of screening detected cases compared to symptom detected cases does not by itself prove any beneficial effects of screening. On the contrary, any screening leading to earlier diagnosis of cancer would be expected to go along with longer survival after diagnosis even if total mortality in the screened and unscreened population remained the same. In such a situation, the apparently longer survival after diagnosis might merely reflect lead time, i.e. advancement of the diagnosis by screening, unless the earlier diagnosis also enhances chances of cure. However, for CRC, chances of cure are strongly stage dependent, and the strong shift of the stage distribution towards earlier stages by colonoscopy or FOBT screening is expected to go along with substantially enhanced chances of cure.

Nevertheless, earlier diagnosis was not the only reason for the substantially enhanced survival of patients with screen-detected cancers because a strong survival advantage of this group persisted even after control for cancer stage and was observed for every stage in stage-specific analyses. Several mechanisms might explain this finding. First, confounding by stage might not be fully accounted for by the relatively crude classification of stage which is an extremely strong predictor of prognosis among CRC patients. However, results remained essentially unchanged in sensitivity analyses specifically adjusting for T- and N-status in addition to UICC stage, and very similar results were obtained after further stratification of stage III cancers according to number of affected lymph nodes. Relevant residual confounding by differences in tumor spread therefore appears unlikely. Second, more slowly growing cancers with more favourable prognosis might have a higher chance to be detected by screening and might be overrepresented in screen-detected cancers compared to symptom-detected cancers, a phenomenon known as “length time bias”. Third, patients adherent to screening recommendations whose cancer was detected by screening might also be more adherent to therapy and might also otherwise behave more health conscious after diagnosis than patients with symptom-detected cancers. However, given that our findings persisted after control for education and given the similarity of non-CRC mortality among screen-detected and symptom-detected cancers, a major role of such “healthy screenee bias” seems unlikely.

Regardless of its origin, our findings of very favourable prognosis of screen-detected cancer cases might have important clinical and public health implications. First, the fear of a fatal diagnosis might prevent many people from using screening offers. Data showing that their prognosis is very good even in the unlikely case that a cancer is found at screening might help to alleviate such fears and enhance adherence to screening recommendations.

Second, direct evidence of relatively favorable prognosis may likewise alleviate fears of patients after a screening initiated diagnosis. Cancer patients are meanwhile often well informed on overall and stage specific cancer survival rates from internet or other information sources. Such survival rates typically do not differentiate between screen-detected and other cancers and may unduly discourage patients with screen-detected cancers. Knowledge of the true prognosis of patients with screen-detected cancers is equally important for the treating physicians and patient-physician interactions, as perspectives of survival and treatment success may impact on treatment decisions. There is ongoing active search for prognostic markers that may support judgment of prognosis as a basis for individual (personalized) treatment decisions. Our results suggest that mode of detection is an exceptionally informative marker in this context which is typically readily available or can be obtained at virtually no extra efforts and costs.

Our study has a number of strengths and limitations. To our knowledge, this is the first study simultaneously assessing and comparing survival outcomes of patients with screening colonoscopy detected CRC, FOBT-detected CRC and symptom-detected CRC, with and without stratification by and adjustment for cancer stage as well as a number of other relevant prognostic factors. Patients were recruited after diagnosis, in most cases during hospital stay or early after discharge. As a result, patients with very early deaths or too sick to participate are likely to be underrepresented. This might have increased observed absolute survival proportions to some extent but should not have affected hazard ratios comparing survival between patient groups. Causes of death were extracted from death certificates which are known to be prone to imprecision and coding errors. However, the validity of recorded cause of death has been consistently found to be much higher for cancers than for other causes of death [26]. Despite the large overall sample size, the limited numbers of deaths in some of the subgroup-specific analyses went along with rather wide confidence intervals. Our data do also not allow disentangling and quantifying the various factors other than earlier stage at diagnosis that might have contributed to the more favorable prognosis of screen-detected cancers. Although we controlled for multiple possible confounders, residual confounding by less than perfectly measured confounders or factors not controlled for cannot be ruled out.

Despite these limitations, our results have important practical implications. Physicians and patients should be aware of the very favorable prognosis after a screen-detected CRC and take this important prognostic factor into account in their treatment decisions. Communication of the favorable prognosis even in the rare case of CRC detection at screening, along with balanced communication of the protective effects [1–11] and the (albeit rare) potential harms of CRC screening, such as bleedings after polypectomy or overdiagnoses [13, 27], might help to enhance acceptance of and adherence to this powerful preventive measure.
